# Pancreatic Cancer—Advances in the Last 50 Years

**DOI:** 10.1002/wjs.70309

**Published:** 2026-04-11

**Authors:** S. George Barreto, Anirban Maitra, Eileen M. O'Reilly, Dushyant Sahani, Richard Zheng, Charles J. Yeo

**Affiliations:** ^1^ Department of Surgery Flinders Medical Centre Adelaide South Australia Australia; ^2^ College of Medicine and Public Health Flinders Health and Medical Research Institute Flinders University Adelaide South Australia Australia; ^3^ Perlmutter Cancer Center New York University Grossman School of Medicine New York New York USA; ^4^ Department of Medicine Memorial Sloan Kettering Cancer Center New York New York USA; ^5^ Weill Medical College at Cornell University New York New York USA; ^6^ University of Washington School of Medicine Seattle Washington USA; ^7^ Jefferson Pancreas, Biliary, and Related Cancer Center Department of Surgery Thomas Jefferson University Philadelphia Pennsylvania USA; ^8^ Department of Surgery Thomas Jefferson University Hospital Philadelphia Pennsylvania USA

**Keywords:** chemotherapy, radiation therapy, staging, surgery, survival

## 1 Background

The last 5 decades have witnessed significant paradigm shifts in the management of pancreatic ductal adenocarcinoma (PDAC) borne by a concerted attempt at understanding its pathogenesis, accompanied by improvements in medical imaging, and a multidisciplinary approach comprising surgery, chemotherapy, and immunotherapy, to guide personalized decision‐making. These advances have translated into modest, but real, improvements in survival and quality of life in recent decades [1]. This special feature article will highlight some of the sentinel events across five domains, namely advances in our understanding of disease pathobiology, imaging, surgery, medical oncology, and pain management. There is a palpable sense of optimism that the knowledge acquired, thus far, will empower us to explore the optimal use of therapies to further improve patient outcomes in the years to come.

## 2 Advances in Imaging

Imaging plays a pivotal role in the early detection, staging, and management of PDAC. Over the past decades, innovations in imaging modalities and computational tools have transformed the diagnosis and management of PDAC. Multidetector CT (MDCT), introduced in the 1990s, remains the cornerstone for staging due to its high spatial resolution and reproducibility [2]. However, modern dual‐energy CT (DECT) is now being preferred for PDAC imaging due to its superior image contrast and lower artifacts, allowing visualization of small or isoattenuating tumors and superior assessment of vascular anatomy [3]. Ghasempourabadi et al. reported DECT sensitivity of 96% and accuracy of 85% for tumor detection and 96% sensitivity with 95% accuracy for nodal staging [4]. Recently introduced photon‐counting detector CT (PCCT) further enhances spatial resolution and tumor conspicuity [5] Table 1.

**TABLE 1:** Summary of imaging modalities and their clinical impact in pancreatic cancer.

Imaging modality	Key advances	Clinical impact
CT (DECT)	High image contrast, artifact reduction	Improved tumor and vascular visualization; accurate staging
Photon CT (PCCT)	Higher image resolution and contrast	Superior tumor conspicuity and tissue characterization
MRI (3T + DWI)	High‐field strength and high quality diffusion‐weighted imaging	Sensitive detection of small lesions and recurrence
PET/CT	Functional and anatomical imaging	Useful in ambiguous cases; moderate accuracy for recurrence
PET/MRI	Simultaneous functional and anatomical imaging	High sensitivity and specificity for recurrence detection
Structured reporting	Standardized lexicons and templates	Improved communication and staging clarity
AI tools	Segmentation, radiomics, predictive analytics	Early detection, risk stratification, personalized planning

Abbreviations: 3T, 3 Tesla; AI, artificial intelligence; CT, computed tomography; DECT, Dual‐energy CT; DWI, diffusion‐weighted imaging; MRI, magnetic resonance imaging; PET, positron emission tomography.

MRI, particularly with 3 T magnets and diffusion‐weighted imaging (DWI), is valuable for screening high‐risk individuals and detecting recurrence. MRI screening in the at‐risk subjects demonstrated 85% sensitivity and 90% specificity [6]. DWI improves early stage disease evaluation and distinguishes recurrence from post‐surgical fibrosis, increasing sensitivity from 61.5% to 88.5% [7].

The adoption of structured reporting templates using standardized lexicons, such as those from the Society of Abdominal Radiology, has had a major impact on improving result communication and staging clarity [8]. Surgeons found surgical planning information significantly more accessible in 60%–98% of structured reports versus 32%–54% of nonstructured reports and sufficient information in 69%–98% versus 25%–43%, respectively [9].

Hybrid imaging modalities like PET/CT and PET/MRI have enhanced functional imaging, aiding in the detection of occult tumors and treatment response assessment. PET/MRI demonstrated 100% sensitivity, 84.6% specificity, and 94.6% accuracy, outperforming PET/CT [10]. Novel PET tracers targeting tumor‐specific markers (e.g., 68Ga‐FAPI) are further improving sensitivity for early disease detection.

In the last decade, AI has emerged as a transformative tool in pancreatic imaging. Deep learning models can detect subtle imaging features predictive of malignancy, even when not visible on the scans. AI‐based extraction of quantitative radiomics features from the pixel data has the potential to support personalized risk stratification and prognostication. Initial studies show AI achieving 90% sensitivity and 93%–96% specificity in detecting pancreatic cancer on CT [11, 12, 13].

These imaging innovations contribute to earlier diagnosis, improved staging, and personalized management of PDAC, aligning with broader multidisciplinary advances in pancreatic cancer care.

## 3 Advances in Pancreatic Cancer Surgery

Pancreatectomy is now considered a prerequisite component of the attempt for the cure of pancreatic cancer. Even as recently as the 1970s, however, postoperative mortality rates exceeded 25%, leading prominent surgeons of the time like Crile and Thorbjarnarson to advocate for abandoning the Whipple entirely [14, 15]. Shortly thereafter, John Cameron and other pioneers proved that it was possible to perform hundreds of pancreatectomies consecutively without any deaths [16, 17, 18]. Today, postoperative mortality is less than 4% and approaches 1% at high‐volume centers [19]. Herein, we celebrate the advances in surgical technique and management that helped usher in this modern era of pancreatic surgery.

**FIGURE 1:**
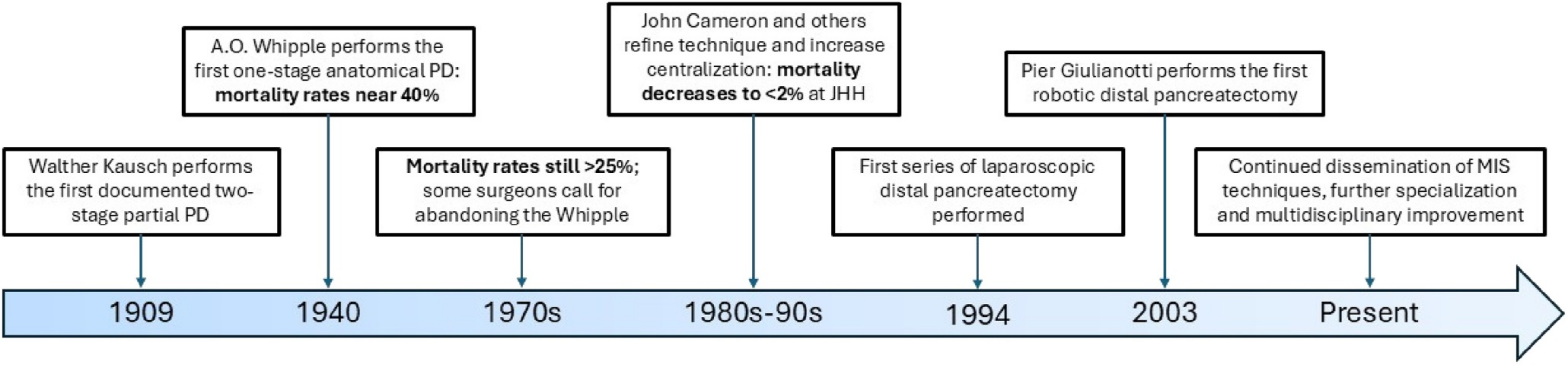
Timeline of major developments in pancreatic surgery. JHH, Johns Hopkins Hospital; MIS, minimally invasive surgery; PD, pancreatoduodenectomy.

This progress was initially made possible by the methodical study of the Halstedian technique through randomized trials (Table 2) [37]. Many landmark trials have focused on mitigating pancreatic fistula, the Achilles heel of pancreatic surgery, and found that techniques such as ductal stenting, fibrin glue, and pancreaticogastrostomy do not improve postoperative pancreatic fistula rates [20, 24, 28]. The prophylactic administration of somatostatin analogs has shown mixed results [31, 38]. Other trials have also demonstrated that fistula rates between duct‐to‐mucosa and invagination pancreaticojejunostomy were equivalent aside from one notable trial which favored invagination [23, 26, 29, 32]. A series of trials from Johns Hopkins have also defined the extent of lymphadenectomy after pancreatoduodenectomy [22, 39, 40]. Several studies have also shown that classic and pylorus‐preserving approaches were largely equivalent with regards to delayed gastric emptying [25, 27, 30]. Other trials have also defined standardized pathways for postoperative care: the HYSLAR trial from Thomas Jefferson showed that hypertonic saline as perioperative maintenance fluid decreased complications [33]. The WARP trial from the same institution also established an accelerated recovery pathway after the Whipple procedure, with a length‐of‐stay targeted at 5 days [34]. Pancreatectomy is now safer than it has ever been and a short postoperative hospitalization is the norm rather than the exception (Figure 1).

**TABLE 2:** Landmark randomized controlled trials advancing pancreatic cancer surgery.

Authors	Year	Location	Major findings
Trials of surgical technique			
Yeo et al. [20]	1995	JHH	Pancreaticogastrostomy does not decrease pancreatic fistula rates
Yeo et al. [21]	2000	JHH	Prophylactic octreotide does not decrease pancreatic fistula rates
Yeo et al. [22]	2002	JHH	Extended lymphadenectomy during whipple does not improve survival
Bassi et al. [23]	2003	Verona	DTM and invagination PJ have similar complication rates
Lillemoe et al. [24]	2004	JHH	Fibrin glue does not decrease pancreatic fistula rates
Tran et al. [25]	2004	Rotterdam	PPPD and classic PD have similar rates of DGE
Langrehr et al. [26]	2005	Berlin	DTM and invagination PJ have similar complication rates
Seiler et al. [27]	2005	Bern	PPPD and classic PD have similar rates of perioperative morbidity
Winter et al. [28]	2006	JHH	Pancreatic duct stenting does not improve pancreatic fistula rates
Berger et al. [29]	2009	TJUH	Invagination PJ is associated with lower pancreatic fistula rates than DTM
Kawai et al. [30]	2011	Wakayama	PD is associated with lower rates of DGE than PPPD
Allen et al. [31]	2014	MSK	Prophylactic pasireotide improves pancreatic fistula rates
El Nakeeb et al. [32]	2015	Mansoura	DTM and invagination PJ have similar pancreatic fistula rates
Trials of postoperative management			
Lavu et al. [33]	2014	TJUH	Perioperative hypertonic saline reduces postoperative complications (HYSLAR trial)
Lavu et al. [34]	2019	TJUH	Early discharge on POD5 is safe and feasible without increasing complications (WARP trial)
Trials of minimally invasive pancreatectomy			
De Rooij et al. [35]	2019	Netherlands	MIDP reduces DGE and time to functional recovery compared to open approach
Klotz et al. [36]	2024	Heidelberg	RPD and open PD have similar morbidity rates

Abbreviations: DGE, delayed gastric emptying; DTM, duct‐to‐mucosa; HYSLAR; JHH, Johns Hopkins Hospital; MIDP, minimally invasive distal pancreatectomy; MSK, Memorial Sloan Kettering; PD, pancreaticoduodenectomy; PJ, pancreaticojejunostomy; POD, post‐op day; PPPD, pylorus‐preserving pancreaticoduodenectomy; RPD, robotic pancreaticoduodenectomy; TJUH, Thomas Jefferson University Hospital; WARP, Whipple accelerated recovery pathway.

Another major area of improvement is in the progress made by our nonsurgical colleagues in avoiding “failure to rescue”. Post‐pancreatectomy hemorrhage was once a rare but often fatal complication [41]. Emergent exploration was traditionally the only option and carried a high mortality rate. Nowadays, however, endovascular embolization or stenting (often of the gastroduodenal artery) by interventional radiologists has supplanted laparotomy and substantially reduced mortality [42]. Interventional radiologists may also help percutaneously drain abscesses and divert bile leaks. Gastroenterologists are similarly capable of endoscopically stenting biliary or pancreatic leaks [43]. Many patients undergoing pancreatectomy are also cared for within specialized surgical intensive care units [44]. These multidisciplinary advances have all contributed to the dramatic improvement in outcomes seen over the last 50 years.

Lastly, pancreatic surgery also continues to evolve and adapt into minimally invasive techniques. Laparoscopic distal pancreatectomy was adopted first and has been associated with faster recovery with equivalent oncologic outcomes [35]. Pancreatoduodenectomy has required the increased dexterity of the robotic platform before being translated into minimally invasive surgery, but robotic Whipple procedures can now be selectively performed by well‐trained surgeons with similar morbidity as compared to the traditional open approach [36, 45, 46, 47, 48, 49]. As the management of pancreatic cancer continues to become more nuanced with more diverse systemic treatments, pancreatic surgery will inevitably develop in tandem.

## 4 Advances in Medical Oncology

### 4.1 Adjuvant and Neoadjuvant Therapy in Pancreatic Cancer

Traditional paradigms for the treatment of localized pancreatic cancer include a surgery first approach for resectable disease. Over the last 20 years, multiple randomized trials have evaluated initially single agent gemcitabine chemotherapy compared to observation following successful surgical resection [50, 51], and more recently, combination chemotherapy regimens, for example, modified(m) FOLFIRINOX (5‐fluorouracil, oxaliplatin, irinotecan, leucovorin), and gemcitabine and capecitabine, compared to gemcitabine have resulted in improvement in disease‐free and overall survival [52, 53, 54]. Although a distinct survival advantage for neoadjuvant systemic therapy followed by surgery compared to surgery first and adjuvant therapy has been demonstrated for borderline resectable PDAC [55, 56], the data for upfront resectable disease have been hard to discern [57, 58, 59]. Multiple randomized trials are underway to address this question; it is very likely that the use of combination chemotherapy in earlier stage PDAC is favorably impacting outcomes by more patients with this disease receiving multimodality therapy, that is, surgery and combination chemotherapy, with the best order in which these therapeutic modalities are delivered being an important point of discussion. Embraced in these improvements in outcome is multidisciplinary discussion and management as part of tumor boards and the access to experienced expertise with this disease [60].

### 4.2 Therapy for Late Stage/Metastatic Pancreatic Cancer

Combination chemotherapy regimens with (*m*) FOLFIRINOX, gemcitabine, nab‐paclitaxel, and NALIRIFOX (liposomal irinotecan, leucovorin, oxaliplatin, 5‐fluorouracil) each have shown improved overall survival, improved progression free survival, and higher response rates compared to the current prior standard of gemcitabine for the first two regimens and to gemcitabine/nab‐paclitaxel in the case of NALIRIFOX [61, 62, 63]. Overall survival has incrementally increased in the last decade and will continue to have impact as these regimens are now routinely used in the setting of localized disease, both locally advanced and resectable pancreatic cancer.

### 4.3 Molecular Biology, Genomic, and Immune Landscape of PDAC to Guide Future Therapy

There have been major improvements in understanding the genomic, both somatic and germline, and immune landscape of pancreatic cancer over the last several decades [64]. Very specifically, the identification of fundamental oncogenic drivers, including *KRAS, TP53, Smad4,* and *Cdkn2a* [65, 66, 67] along with other rarer genomic alterations, have provided major insights into the pathobiology and therapeutic opportunities for this disease. About 5%–10% of pancreatic cancers have a germline hereditary predisposition, including *BRCA*1/2*, PALB2, ATM,* and the various Lynch genes (*MLH1, MSH2 MSH6, PMS2*) that may have therapeutic actionability and can lead to the identification of a family at risk for other diseases and hence screening and early detection opportunities [68, 69], although the impact of screening specifically for pancreatic cancer even in genomically enriched subgroups can be challenging to discern and may result in detection at an earlier stage. For germline *BRCA1/2*, a maintenance poly‐ADP‐ribose polymerase inhibitor, Olaparib [70], demonstrated significant improvement in progression free survival following platinum‐based therapy, compared to observation/placebo in metastatic PDAC. Further, the therapeutic potential of *Kras* mutations being near ubiquitous in pancreatic cancer and alterations in other parts of the mitogen activated kinase pathway (MAPK) in nearly 95% of individuals with this disease are about to be realized. KRAS inhibitors have entered the clinic, and early promising signals have been identified for the 1% of individuals with pancreatic cancer and *Kras* G12C mutations, and late‐stage clinical trial data are anticipated in 2026, evaluating a panRAS inhibitor which has an activity in the more common KRAS alleles of *Kras* G12D, G12V, and G12R compared to standard combination chemotherapy in previously treated metastatic pancreatic cancer. These drugs, both panRAS and allele specific agents, are moving quickly in development to an earlier stage disease. For the small subset of individuals with no *Kras* mutation (*KRAS* wild‐type), there are targeted therapeutics that impact various signaling pathways, including *NRG‐1* [71]*, NTRK, BRAF, ROS, ALK,* and others, and can have a meaningful impact on the disease. Specifically, the first bispecific in pancreatic cancer, zenocutuzumab [71], received a disease‐specific regulatory approval in 2024. Similarly, for the small subset of individuals with mismatch repair deficiency/microsatellite instability (dMMR, MSI‐H), immune checkpoint inhibitors have demonstrated a meaningful value in this disease. Collectively, the value and impact of precision‐based medicine approaches have resulted because of routine germline testing and somatic next generating sequencing in this disease. Newer tools, including circulating tumor DNA (ctDNA) and RNA sequencing profiling, will further augment the value and identification of newer targets. Traditionally, pancreatic cancer has been generically considered as the prototype immunologically cold disease; however, the recognition that the treatment of this disease with neoantigen and oncogene targeted approaches can generate a potent immune response in subsets of individuals provides optimism for the coming decade [72, 73].

## 5 Advances in Supportive Measures, Including Management of Pain

In parallel with these above stated developments has been the moving away from a perspective of therapeutic nihilism and for more patients diagnosed with this disease having the option to consider treatment options as opposed to nonreferral. In the fields of gastroenterology and interventional radiology effective palliation of malignant biliary obstruction and duodenal/gastric outlet obstruction by the use of endoscopic [74] and percutaneous stenting can meaningfully impact symptomatology in this disease. In parallel, usage of pancreatic enzyme replacement therapy [75] to treat pancreatic exocrine insufficiency that accompanies PDAC [76], effective pain palliation [77] including the use of chemical splanchnicectomy [78]/celiac plexus neurolysis within a multidisciplinary team approach can optimize symptom control and improve both quality and length of life.

## 6 Advances in Pancreatic Cancer Pathobiology

### 6.1 Genetically Engineered Mouse (GEM) Models for Studying Pancreatic Cancer in the Laboratory

Although the pancreas was the first organ where transgenesis was successfully accomplished in 1984 [79], the early GEM models of pancreatic cancer did not resemble the multistep progression of ductal adenocarcinoma, preceded by intraductal PanIN lesions, observed in humans [80, 81]. In 2003, this barrier was successfully overcome through GEM models that expressed mutant *Kras* from the endogenous locus in the exocrine pancreas within the transcriptional domain of *Pdx1* and later *Ptf1/p48* [82]. These data confirmed the seminal role of mutant Ras in the initiation of exocrine pancreatic neoplasia, with cooperating driver alterations (*Smad4*, *Cdkn2a/p16*, *p53*) essentially altering the penetrance and natural history of this Ras‐induced disease process [83, 84, 85]. Subsequently, animal models where mutant *Kras* can be induced by doxycycline were developed [86, 87], which confirmed that the genetic extinction of Ras in established tumors had the potential to attenuate tumor growth. In many respects, these genetic studies provided the compelling rationale for pharmacological targeting of KRAS in PDAC, which has now become a reality in the past few years [88]. More recently, dual recombinase models have also been developed which have allowed for asynchronous perturbation of genes within the epithelial and stromal compartments, respectively [89, 90, 91]. In addition to models of PDAC that recapitulate the more common noncystic pathway to invasive cancer, mice that develop cystic lesions resembling intraductal papillary mucinous neoplasms have also been generated [92, 93, 94]. Cumulatively, the development of these autochthonous models has led to significant developments in pancreatic cancer research, from mechanistic understanding of cancer cell intrinsic biology [95, 96, 97, 98, 99], the host (stromal and immune) response to multistep tumorigenesis [100, 101, 102, 103], and the influence of environmental influences like diet and exercise [104, 105, 106]. Further, these models are widely used in evaluating experimental therapies prior to human translation [107, 108]. Although orthogonal preclinical models like murine and patient‐derived organoids have been invaluable in accelerating translational research [109, 110], one can argue that GEM models represent one of the pivotal advances in this field with a lasting impact.

### 6.2 Deconstructing Tumor and Host Heterogeneity With Single Cell RNA and Spatial Profiling

Prior to the advent of dissociative single cell RNA sequencing (scRNA seq) and, more recently, spatial transcriptomics (ST), it was commonly believed that pancreatic cancers were relatively homogeneous, and that the host response was monolithic. This viewpoint is now obsolete, with scRNA‐seq and ST approaches underscoring the profound tumor and host heterogeneity that characterizes this disease [111, 112]. Although the full scope of discussion of PDAC heterogeneity is outside the scope of this commentary, a couple of vignettes will be highlighted. First, we have now recognized that the entity “cancer associated fibroblasts” or CAFs is an admixture of multiple CAF subtypes including myofibroblastic CAFs (myCAFs), inflammatory CAFs (iCAFs), and antigen presenting CAFs (apCAFs) [113, 114, 115]. Each CAF subtype can be defined by distinct expression patterns on RNA and proteomics analyses [116, 117], as well as distinct cellular neighborhoods within the tumor microenvironment in which they can be found [118, 119]. Further, we have also recognized the divergent roles of CAF subtypes in promoting or restraining tumor progression [120]. Second, we have also recognized that beyond genetic heterogeneity, PDAC cells are characterized by transcriptomic heterogeneity with the recognition of at least two major cell states (classical and basal‐like) [121, 122] and additional states defined based on further refinement of profiling data [123]. Recent spatial data have also highlighted the profound intra‐tumoral heterogeneity of epithelial lineage states in primary and metastatic tumors [124], as well as the susceptibility to distinct therapeutic regimens (such as chemotherapy vs. targeted therapies like KRAS inhibitors) [125, 126]. The recognition of the cancer cell and stromal heterogeneity will continue to have profound effects on how more efficacious and selective therapeutic regimens are designed for this disease.

In summary, the future for treatment of PDAC is brightly related to a renewed focus on this recalcitrant cancer, recognition of the importance of the value of multiagent cytotoxic chemotherapy for all disease stages, integration of targeted therapy options where feasible and indicated and recognition of the value and importance of multidisciplinary care. Leveraging RAS targeting and capitalizing on novel immune therapeutics are further poised to improve outcomes in PDAC.

## Author Contributions

**S. George Barreto:** conceptualization, project administration, data curation, investigation, methodology, formal analysis, writing – original draft, writing – review and editing. **Anirban Maitra:** data curation, investigation, methodology, formal analysis, writing – original draft, writing – review and editing. **Eileen M. O'Reilly:** data curation, investigation, methodology, formal analysis, writing – original draft, writing – review and editing. **Dushyant Sahani:** data curation, investigation, methodology, visualization, formal analysis, writing – original draft, writing – review and editing. **Richard Zheng:** data curation, investigation, methodology, visualization, formal analysis, writing – original draft, writing – review and editing. **Charles J. Yeo:** data curation, investigation, methodology, visualization, formal analysis, writing – original draft, writing – review and editing.

## Funding

The authors have nothing to report.

## Conflicts of Interest

Eileen M. O'Reilly received Research Funding to Institution: Genentech/Roche, BioNTech, AstraZeneca, Arcus, Elicio Therapeutics, Parker Institute, NIH/NCI, Digestive Care, Break Through Cancer, Agenus, Amgen, Revolution Medicines; Consulting/DSMB: Uncompensated: Arcus, Amgen, AstraZeneca, Ability Pharma, Alligator BioSciences, Pfizer, Agenus, BioNTech, Ipsen, Ikena, Merck, Immuneering, Moma Therapeutics, Novartis, Astellas, BMS, Revolution Medicines, Regeneron, Tango Therapeutics; Travel: BioNTech, Arcus, Other: American Association of Cancer Research (AACR), American Society of Clinical Oncology (ASCO), Imedex, Research To Practice, Stand Up To Cancer (SU2C), NIH/NCI. The other authors declare no conflict of interest

## Data Availability

The authors have nothing to report.
